# Generation and Degeneration of the Tissues of the Mouth

**Published:** 1895-10

**Authors:** W. H. Whitslar

**Affiliations:** Cleveland, O.


### Generation and Degeneration of the Tissues of the Mouth.

BY W. H. WHITSLAR, M.D., D.D.S., CLEVELAND, O.

Read before the Tri-State Dental Meeting at Detroit, June 18th, 1895.

The first general observation we should perceive in the study of vital phenomena is the mutual dependence of all things, which in their groupings together compose the system of Nature. This dependence requires for each being the existence of all the others.

Anatomy is a part and parcel of physiology, and, of all studies, it enables us to obtain the best appreciation of the beauties of living phenomena. It is essential to understand the situation, size, form and structure of organs, and their relations, in order to have a perfect conception of their functions.

Now, the phenomena of development, growth, sensation, decay and death, and many others, belong to life. Life occurs only in material structures which exist in obedience to the laws of physics and chemistry, and is a superstructure upon these laws which cannot be studied independently of them. Indeed, the greater part of the phenomena of organic beings is chemical and physical, modified only by an additional principle called life.

Man's life is inseparably linked with the plants and animals, and his existence depends upon the climate and productions of the earth. His own organic constitution participates in all things that surround him, hence it is necessary, in order to understand the conditions of our existence, to begin at the germ of organization and pursue the changes that occur in the nearest approximation to the inorganic material of the universe. We should study the protozoa, and rise in the scale until we arrive at the height of God's perfected being—man. In this study we would find the mutual dependence of all living organisms, and the expanse of power widening as the scale of organization ascends, each being independent in propogation of species. Whilst there is a vital power given to each organism in the first place by the Creator, there is a correllated physical and chemical relationship thereafter between individuals, in accordance with their environments. Recognizing the dependence of man upon his surroundings and his predecessors, in a measure, his individualism is born when the pronucleus of the ovum is impregnated with the spermatazoon.

If the theory of Weissman be true, then in this microscopic cell there exists germ plasm that has been transmitted, or a continuity is established that carries with it the natural impress of parental characteristics. In this way heredity forms an active agent in the generation of all parts of the economy, and it is an important etiological factor in pathogeny.

So, beginning with the cell, or egg, its first aptitude is its adaptation to its environments, and in the hope of the establishment of an equilibrium between itself and parent it extracts from its trysting place pabulum until it is expelled from the body. The equilibrium between parent and embryo is not recognized until the child is entirely independent, and a personal existence commences.

To be specific, we must particularize. Every animal arises from an egg, i. e., an impregnated cell from the female. The union of this protroplasmic material involves chemical and physical phenomena, and, endowed with united vital forces, organization is promoted. These chemical changes increase in proportion to the growth. Indeed, the cell is the simplest physiological apparatus, and as such is the seat of chemical processes. It is generally conceded that all chemical changes of importance do not take place in the animal fluids, but that these occur in the cells. The cells regulate by their activities the chemical processes and exchange of foods.

In the generating of the tissues of the mouth from the primitive cell, the process of segmentation evolves the ectoderm and entoderm, from which springs the mesoderm. Just why these layers of cells, which are seemingly evolved from the same material, should mature different tissues is difficult to understand. All we can say is that there is a different chemical and physical arrangement of the molecules of matter presided over by existing vital forces.

That vital phenomena have to do with the chemical arrangement of bodies is exemplified by the experiments of Mr. Rainey in the processes of calcification. In the formation of enamel or dentine, calcific material is held in chemical combination with the intercellular substances in the vicinity of, and in, the enamel organ and dentinal papilla. By the action of ameloblasts and odontoblasts—specialized cells—enamel and dentine are formed. It is by their activities that the lime is deposited chemically as well as physically.

An aggregation of cells composes what is termed the enamel organ, and it is said that the function of the enamel organ is the formation of enamel. Strictly speaking, however, it is a matrix to mold the form of the tooth, the performance of function being resident in the ameloblasts.

Function is a vital phenomenon: and pathologic conditions are perverted physiological phenomena. The difference between these two conditions is an indefinite line wherein the balance of vitality is overcome by perverted function. If tissues are degenerated from their normal tendencies, regeneration balances the waste, but if they are normal it is a physiological process. If, however, the cell destruction is greater than the cell production, regeneration is suspended and atrophy results. Thus we observe that the degree of atrophy is proportionate to the diminution of function.

Now we find frequently that teeth have white and brown spots that are congenital. These spots are the result of perverted function of the cells that superintend the deposition of the lime salts. This perversion is ostensibly caused by starvation or improper metabolism of foods. It would not be entirely amiss to surmise that the white spots were produced by increased function, but limited in the supply of organic materials. In these we find the intermediary organic substance is deficient. The colored spots contain a greater amount of organic matter than the white spots. This may be due to the sluggishness of circulating fluids in the vicinage, resulting in stagnation or lessening of the chemical and physical processes. Thus, while other parts of the tooth are developing, an area of imperfect tooth material is constructed, which is due to the lack of power of the cells which have to do with the building, so to speak. There is a failure of correllation of the organic and inorganic substances, and the tooth becomes more of an admixture than a proper organization.

Notwithstanding criticisms to the contrary, I believe that hæmoglobin and oxyhæmoglobin of the blood and their derivatives, hæmochromogen, hæmatin and methæmoglobin, acting as transudation products, or even the results of decompositions, stain these areas by being incorporated with the material.

It is to be remembered that the blood vessels are in close proximity to the ameloblasts, also that these cells are derived from the malpighian layer of cells, which have to do with the pigmentation of the mucous membrane. There is in this a close analogy which may be significant. However, we must rely upon the activities of the blood in the vicinity, for it is the first tissue to exhibit extreme atropic changes.

It is necessary to have blood present after the first stages of calcification to further that process. During these preliminary stages the mineral elements may be found in proximity to the developing tooth, and from these the primary deposits may be accrued.

The mineral substances are the most essential constituents of the teeth. Phosphate of lime is greater in amount in the teeth than any other tissue. The consumption of it during pregnancy is often so great that it does not appear in the usual amounts in the excreta, scarcely any trace being found (Lehman). This may account in part for Lehman's declaration that lime salts are deposited mechanically in bones. He gives as proof, the ease with which lime salts can be so thoroughly dissolved from bones by hydrochloric acid. Acting upon this hypothesis, may we ask the question, "Are enamel and dentine deposited mechanically?"

This is partially true, I believe, for, as already stated, there is resident material in the part, as well as that which is carried to it by blood and intercellular fluids. This may be a process of osmosis, or it may be one of catalysis. We learn that "earthy phosphates are found in all cells and tissues. Indeed, they seem to be of greatest importance for the life of the cells and the chemical processes that accompany their evolution; that it is impossible to separate them from the protein substances without decomposition. There is no animal tissue which does not contain mineral substances." "The bones, teeth and muscles contain the most minerals." "In the distribution of mineral substances we find them dissolved in the fluids and partly combined with organic substances."

The question naturally arises, are mineral substances manufactured within the body? It is established that on the burning of the organic substances the mineral bodies are liberated and eliminated. They, in part, combine with new products of the oxidation and become attached to the organic bodies which are free from salts and are absorbed from the intestinal canal.

Hence, it would be seen that a constant supply of mineral substances is not absolutely necessary, and that an insignificant amount of inorganic bodies must be administered. So I wish to argue that, for this scientific reason alone, it is not a necessity to administer constantly foods bearing large quantities of phosphatic materials to develop good teeth. We must rely upon the resiliency of life as the energizing power to create metabolism. Physical forces strive to maintain themselves in equilibrium—thus we have metabolic power. A point of rest, normal state of being, is attained because physical forces act upon matter even if it has attained its equilibrium. Inorganic chemistry also induces motion, and continues active in motion and metamorphoses until the closest affinities are satisfied. Albinus established the axiom that the essence of vital force consisted in motion. But if that vital power is by disease deficient, then the metabolism of materials into tooth structure is obstructed, and we see the results in deformed teeth, both as to form and structure.

Let us, for example, suppose that fever disturbs the parturient woman, she is robbed of nutritive power to supply her embryo. Unfortunately Nature does not come to the rescue and, the equilibrium being unbalanced, she is sacrificed at the expense of her general health, and the embryo develops, but less actively. Because of this sacrifice we seldom witness defective deciduous teeth, and unless the parent recuperates, the disease shows its tracings upon the permanent teeth; in other words, produces a disease destroying the pivotal anchorage upon which the balance of vital phenomena is supported.

Rachitis and concomitant diseases, affecting particularly the bony structures, are resultant from imperfect metabolism and starvation. The mineral substances leave the body uninterruptedly in starvation until death (Hammarsten). The experiments of Chossat and Voit show the loss of weight of bones during starvation to be as high as seventeen per cent. in pigeons, and fourteen per cent. in cats. Blood and its solid ingredients decrease in proportion to the weight of the body. Naturally, then, the teeth, whose development is dependent upon blood, must suffer.

It is not necessarily sufficient to argue that the teeth suffer in the same proportion as bones, because one system of organs may derive its nutriment at the expense of another organ, so it is impossible to say that teeth receive their aid from this or that. They do suffer, however, and all the permanent teeth developing whilst in utero show at times marked signs of starvation. The cells, which are the constructive agents of the the teeth, may have such environments that the resiliency of their activity is sufficient to controvert the disease: their chemical and physical activities are alert, and extract from other tissues material for a continuance of construction. This seems to be a gift of specialized cells.

Phosphate of lime is an important adjunct in metamorphosis of animal tissues. We receive much of it through our food. The graminivorous animal receives it through the vegetable kingdom in certain nitrogenous bodies which contain phosphate of lime, as in vegetable albumin, legumin and glutin. Phosphate of lime is not removed from the body until it is partially decomposed or oxidized, and in this process phosphoric acid must accrue, which enters into union with the lime that enters the body with cereals and leguminous plants. Hence, we observe the relation of our bodies to all that surrounds us, as stated in the beginning of this paper. Our body is a great chemical laboratory, in which wonderful phenomena are exhibited.

Developing tissues of the mouth have great reliance upon chemism, and improper construction may be due to the disassoiaction of molecules of matter, all these being modified by the principle of life.

